# Supplementary material for: Mitigation bank applications for freshwater systems: Control mechanisms, project complexity, and caveats
Source: PLoS One. 2024 Feb 6;19(2):e0292702. doi: 10.1371/journal.pone.0292702 (PMC10846733; doi:10.1371/journal.pone.0292702)
Supplement: S1 Table — Performance and assessment aspects used by the three bank types. (DOCX) [file pone.0292702.s001.docx]

**Table S1. Bank monitoring.** Performance and assessment aspects used by the three bank types.

| **Performance and assessments aspects** | **CBR**  **(n = 22)** | **PAC**  **(n = 14)** | **HCS**  **(n = 11)** |
| --- | --- | --- | --- |
| **Hydro/Geomorphology** | | | |
| Erosion and stability | 68.1% (15) | 50% (7) | 36.4% (4) |
| Flow | 100% (22) | 78.6% (11) | 54.5% (6) |
| Hydrology | 100% (22) | 64.3% (9) | 100% (11) |
| **Protection and control** | | | |
| Invasive species control | 40.9% (9) | 50% (7) | 81.8% (9) |
| Maintenance and access | 100% (22) | 28.6% (4) | 54.5% (6) |
| **Fish** | | | |
| Species abundance | 36.4% (8) | 35.7% (5) | 81.8% (9) |
| Species biomass | 13.6% (3) | 28.6% (4) | 36.4% (4) |
| Species presence | 100% (22) | 50% (7) | 81.8% (9) |
| Spawning evidence | 18.1% (4) | 14.3% (2) | 54.5% (6) |
| Diet | - | - | 36.4% (4) |
| Community structure | - | - | 36.4% (4) |
| **Riparian buffer** | | | |
| % Cover (vegetation) | 9.1% (2) | 35.7% (5) | 63.6% (7) |
| Stem count (vegetation) | - | 28.6% (4) | 54.5% (6) |
| % Survival (vegetation) | - | 50% (7) | 72.7% (8) |
| Shade (%) | 13.6% (3) | 14.3% (2) | 54.5% (6) |
| **Biochemical** | | | |
| Water quality | 9.1% (2) | 50% (7) | 72.7% (8) |
| Soil sampling | 22.7% (5) | 28.6% (4) | 54.5% (6) |
| **Habitat features** | | | |
| Aquatic cover (%) | 9.1% (2) | 28.6% (4) | 63.6% (7) |
| Spawning and rearing (number) | - | 28.6% (4) | 54.5% (6) |
| Structural integrity (%) | - | 35.7% (5) | 36.4% (4) |
| Substrate (type & dominance) | 13.6% (3) | 28.6% (4) | 54.5% (6) |
