# Supplementary material for: Mitigation bank applications for freshwater systems: Control mechanisms, project complexity, and caveats
Source: PLoS One. 2024 Feb 6;19(2):e0292702. doi: 10.1371/journal.pone.0292702 (PMC10846733; doi:10.1371/journal.pone.0292702)
Supplement: S2 Table — Generalized Additive Model output for bank type monitoring and release schedule timeframes over bank complexity for CBR banks. (DOCX) [file pone.0292702.s002.docx]

**Table S2. CBR banks model output.** Generalized Additive Model output for bank type monitoring and release schedule timeframes over bank complexity for CBR banks.

| Bank-type  (CBR) | Estimate Std. | Error | t-value | Pr(>\|t\|) |
| --- | --- | --- | --- | --- |
| Intercept | 7.000 | 0.364 | 19.23 | 6.6e-14 |
|  | edf | Ref.df | F | p-value |
| Monitoring time | 2.017 | 2.443 | 1.259 | 0.442 |
|  | k’ | edf | k-index | p-value |
|  | 4.00 | 2.02 | 1.02 | 0.47 |
|  | Estimate Std. | Error | t-value | Pr(>\|t\|) |
| Intercept | 3.7273 | 0.2627 | 14.19 | 6.66e-12 |
|  | edf | Ref.df | F | p-value |
| R-Schedule | 1 | 1 | 0.006 | 0.94 |
|  | k’ | edf | k-index | p-value |
|  | 4 | 1 | 1.14 | 0.66 |
