# Supplementary material for: Mitigation bank applications for freshwater systems: Control mechanisms, project complexity, and caveats
Source: PLoS One. 2024 Feb 6;19(2):e0292702. doi: 10.1371/journal.pone.0292702 (PMC10846733; doi:10.1371/journal.pone.0292702)
Supplement: S3 Table — Generalized Additive Model output for bank type monitoring and release schedule timeframes over bank complexity for PAC banks. (DOCX) [file pone.0292702.s003.docx]

**Table S3. PAC banks model output.** Generalized Additive Model output for bank type monitoring and release schedule timeframes over bank complexity for PAC banks.

| Bank-type  (PAC) | Estimate Std. | Error | t-value | Pr(>\|t\|) |
| --- | --- | --- | --- | --- |
| Intercept | 9.786 | 1.015 | 9.642 | 5.3e-07 |
|  | edf | Ref.df | F | p-value |
| Monitoring time | 1 | 1 | 1.338 | 0.27 |
|  | k’ | edf | k-index | p-value |
|  | 4 | 1 | 1.28 | 0.8 |
|  | Estimate Std. | Error | t-value | Pr(>\|t\|) |
| Intercept | 5.7857 | 0.4262 | 13.57 | 1.21e-08 |
|  | edf | Ref.df | F | p-value |
| R-Schedule | 1 | 1 | 5.438 | 0.0379 |
|  | k’ | edf | k-index | p-value |
|  | 4 | 1 | 1.27 | 0.74 |
