# Supplementary material for: Mitigation bank applications for freshwater systems: Control mechanisms, project complexity, and caveats
Source: PLoS One. 2024 Feb 6;19(2):e0292702. doi: 10.1371/journal.pone.0292702 (PMC10846733; doi:10.1371/journal.pone.0292702)
Supplement: S4 Table — Generalized Additive Model output for bank type monitoring and release schedule timeframes over bank complexity for HCS banks. (DOCX) [file pone.0292702.s004.docx]

**Table S4.** **HCS banks model output**. Generalized Additive Model output for bank type monitoring and release schedule timeframes over bank complexity for HCS banks.

| Bank-type  (HCS) | Estimate Std. | Error | t-value | Pr(>\|t\|) |
| --- | --- | --- | --- | --- |
| Intercept | 12.3636 | 0.9959 | 12.41 | 5.77e-07 |
|  | edf | Ref.df | F | p-value |
| Monitoring time | 1 | 1 | 3.697 | 0.0867 |
|  | k’ | edf | k-index | p-value |
|  | 4 | 1 | 1.47 | 0.88 |
|  | Estimate Std. | Error | t-value | Pr(>\|t\|) |
| Intercept | 7.3636 | 0.4293 | 17.15 | 3.5e-08 |
|  | edf | Ref.df | F | p-value |
| R-Schedule | 1 | 1 | 6.068 | 0.036 |
|  | k’ | edf | k-index | p-value |
|  | 4 | 1 | 1.43 | 0.86 |
