# Supplementary material for: Mitigation bank applications for freshwater systems: Control mechanisms, project complexity, and caveats
Source: PLoS One. 2024 Feb 6;19(2):e0292702. doi: 10.1371/journal.pone.0292702 (PMC10846733; doi:10.1371/journal.pone.0292702)
Supplement: S5 Table — ANOVA output for the number of credits (%) released over different bank stages and pairwise comparisons for bank types. (DOCX) [file pone.0292702.s005.docx]

**Table S5. ANOVA table.** ANOVA output for the number of credits (%) released over different bank stages and pairwise comparisons for bank types.

| Credits | Df | Sum Sq | Mean Sq | F-value | Pr(>F) |
| --- | --- | --- | --- | --- | --- |
| Stage | 3 | 13487.0 | 4495.7 | 31.834 | < 2.2e-16 |
| Type:Stage | 6 | 9070.9 | 1511.8 | 10.705 | 3.918e-10 |
| Residuals | 176 | 24854.6 | 141.2 |  |  |
| Stage 1 | | CBR | | HCS | |
| HCS | | 0.020 | | - | |
| PAC | | 0.044 | | 0.647 | |
| Stage 2 | | CBR | | HCS | |
| HCS | | 0.023 | | - | |
| PAC | | 0.422 | | 0.148 | |
| Stage 3 | | CBR | | HCS | |
| HCS | | 0.034 | | - | |
| PAC | | 0.438 | | 0.188 | |
| Stage 4 | | CBR | | HCS | |
| HCS | | 9.2e-08 | | - | |
| PAC | | 0.0038 | | 0.0071 | |
