# Supplementary figures and images for: Mitigation bank applications for freshwater systems: Control mechanisms, project complexity, and caveats
Source: PLoS One. 2024 Feb 6;19(2):e0292702. doi: 10.1371/journal.pone.0292702 (PMC10846733; doi:10.1371/journal.pone.0292702)

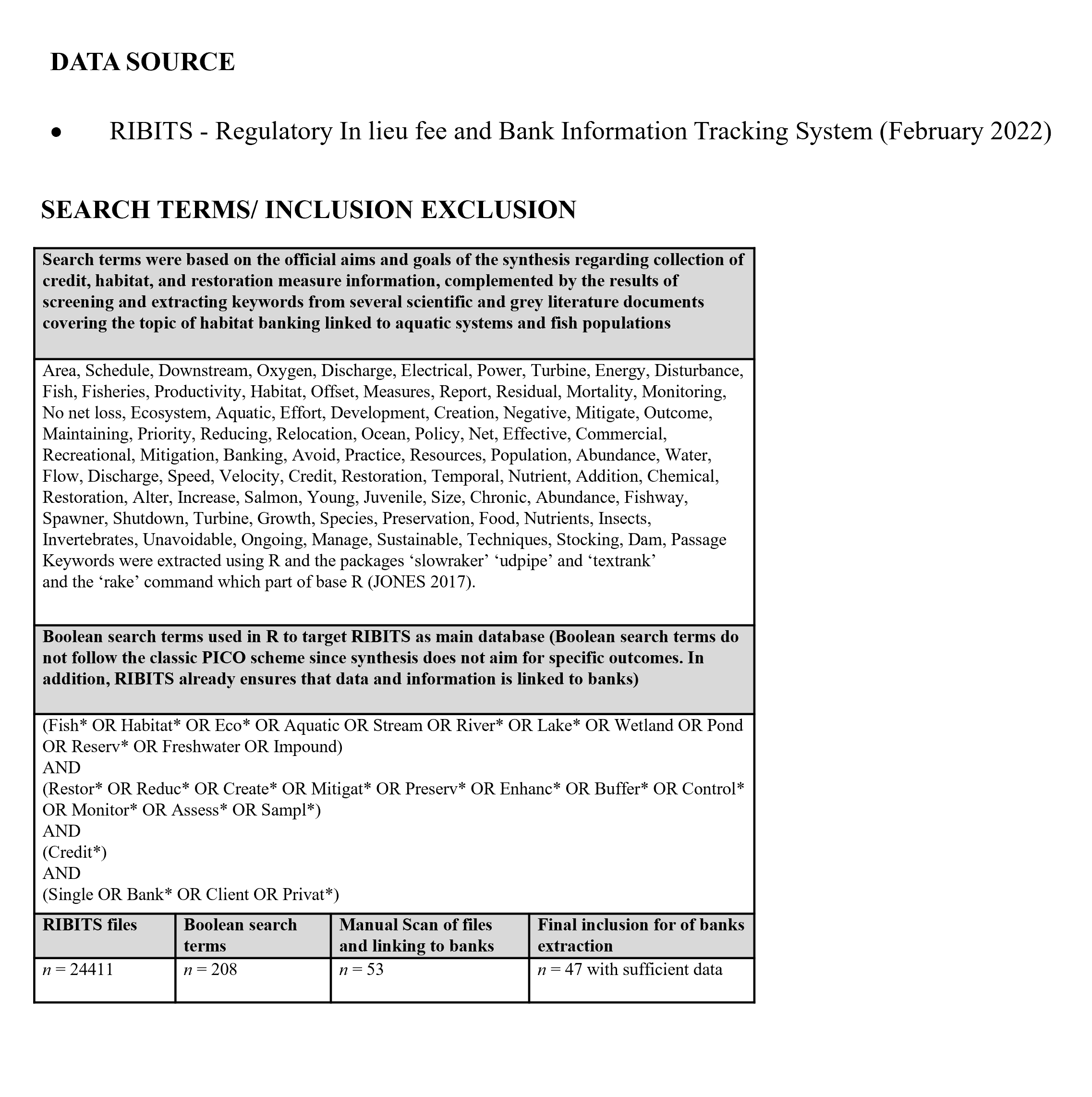

Supplement: S2 Fig — (TIF) [file pone.0292702.s007.tif]
