# Supplementary material for: Mitigation bank applications for freshwater systems: Control mechanisms, project complexity, and caveats
Source: PLoS One. 2024 Feb 6;19(2):e0292702. doi: 10.1371/journal.pone.0292702 (PMC10846733; doi:10.1371/journal.pone.0292702)
Supplement: Attachment — Submitted filename: Reviews_comments.docx [file pone.0292702.s008.docx]

PONE-D-23-15042

Mitigation bank applications for freshwater systems: Control mechanisms, project complexity, and caveats

PLOS ONE

[Note: HTML markup is below. Please do not edit.]

**We would like to thank Reviewers for taking the time and effort necessary to review the manuscript. We sincerely appreciate all valuable comments and suggestions, which helped us to improve the quality of the manuscript.**

Reviewers' comments:

Reviewer's Responses to Questions

**Comments to the Author**

1. Is the manuscript technically sound, and do the data support the conclusions?

The manuscript must describe a technically sound piece of scientific research with data that supports the conclusions. Experiments must have been conducted rigorously, with appropriate controls, replication, and sample sizes. The conclusions must be drawn appropriately based on the data presented.

Reviewer #1: Partly

**Reply: Thank you for your comment.**

**As a quick overview here are the main changes done to the manuscript to improve technical soundness:**

**1. Inclusion of extended literature review to highlight the knowledge gap better.**

**2. Simplified overview and explanation on how mitigation banking/ offsetting works so it should be more accessible for people outside of the field (E.g., Figure 1).**

**3. Extension and rework of the method section to explain variables better and provided a step-by-step walkthrough of each variable and its role (E.g., Table 1 & 2).**

**4. Walkthrough example for a bank (Figure 3).**

**5. Extension of provided method explanations e.g., better justification for GAMs etc. in the method section.**

**6. Conclusion section with main take home messages and high-level recommendations.**

2. Has the statistical analysis been performed appropriately and rigorously?

Reviewer #1: No

**Reply:**

**Thank you for your comment. Would you be able to specify which part of the analysis has not been performed appropriately or rigorously? There might have been some confusion based on the lengthy method section as well as combining variable descriptions and statistical approaches. Hence the section has been restructured to better reflect variable content and statistical measures separately. We also want to reiterate the use of supplements as e.g., Figure S1 holds much of the conceptual and analytical framework and should be used in conjunction with reading the manuscript. Please refer to the updated method section. If not here is a short summary on how things have been changed:**

**2.1 Still the same and covers basic data acquisition which should be intuitive.**

**2.2-2.4 Formerly key variables have been changed to two different full tables (1 & 2) that hold all qualitative and quantitative variables with description and use. This should address your concerns and help explain how they are used in their own respective way to answer the individual research questions. This also frees up space in the method section to justify and support variable use more.**

**We also acknowledge that mitigation banking as well as offsetting is a still rather novel field with which not everyone is familiar in the environmental sector. Thus, we have added a new Figure 1 & 3 to the Introduction/ Methods to include a simplified approach on how mitigation banking works – a) is an example of traditional offsetting and b) the third-party introduction of a bank. Figure 3 is a walkthrough of calculating scores for an example bank.**

**2.5 Extension of used statistical analyses to explain and justify the use of GAMs better.**

3. Have the authors made all data underlying the findings in their manuscript fully available?

The [PLOS Data policy](http://www.plosone.org/static/policies.action#sharing) requires authors to make all data underlying the findings described in their manuscript fully available without restriction, with rare exception (please refer to the Data Availability Statement in the manuscript PDF file). The data should be provided as part of the manuscript or its supporting information, or deposited to a public repository. For example, in addition to summary statistics, the data points behind means, medians and variance measures should be available. If there are restrictions on publicly sharing data—e.g. participant privacy or use of data from a third party—those must be specified.

Reviewer #1: Yes

**Reply: -**

4. Is the manuscript presented in an intelligible fashion and written in standard English?

PLOS ONE does not copyedit accepted manuscripts, so the language in submitted articles must be clear, correct, and unambiguous. Any typographical or grammatical errors should be corrected at revision, so please note any specific errors here.

Reviewer #1: No

**Reply:**

**Draft has been reviewed by native English speakers as well as grammar software. If you still feel that it is unclear, please specify the nature of your concerns in whether they refer to accessibility, comprehensiveness, or grammar/ spelling. Certain terms are inherently hard to describe outside the context of mitigation banking and might not be fully accessible or translatable to ley terms.**

5. Review Comments to the Author

Please use the space provided to explain your answers to the questions above. You may also include additional comments for the author, including concerns about dual publication, research ethics, or publication ethics. (Please upload your review as an attachment if it exceeds 20,000 characters)

Reviewer #1: Introduction
- Include literature gap and study’s contribution. Apart from giving a good background, the introduction must reveal the existing literature gap and consequently what your study contributes to. Also, please, kindly read the instructions for the authors to be abide by the requirements of the journal. You might not need to isolate the literature review as a separate section, it could be part of the introduction in different paragraphs.
Material and methods
- The authors need to present the analytical framework in detail supported by literature, define and describe the variables used in the study and explain the model employed.
- Try to be more specific and clearer of variables measurement!
Results and discussion
- Please read the author’s guide and appropriately develop this section.
Conclusion
- The conclusion should be empirically supported by the results and should give conclusion on aspects presented in the results section. Authors must bear in mind that conclusion is simply a ‘take home message’ based on the findings presented. Therefore, I strongly suggest that authors reconstruct the write-up in the conclusion and include policy recommendations.

**We have extended the reviewed studies in the introduction to focus on the key available reviews covering offsetting and mitigation. We acknowledge that there are more studies out there, especially in a regional context but the mentioned ones (~50) are the keystone studies and papers that have attempted system wide and large-scale reviews and syntheses of the mitigation sector and framework.**

**Conclusions with take home messages have been synthesized from the discussion and added to the manuscript to increase accessibility.**
